# Clinical characteristics and outcome of HIV infected patients with chronic kidney disease in Sub Saharan Africa: an example from in Cameroon

**DOI:** 10.1186/s12882-019-1446-3

**Published:** 2019-07-09

**Authors:** Marie Patrice Halle, Noel Essomba, Hilaire Djantio, Germaine Tsele, Hermine Fouda, Namme Henri Luma, Enow Gloria Ashuntantang, Folefack Francois Kaze

**Affiliations:** 1 Faculty of Medicine and Pharmaceutical Sciences, University of Douala, Douala, Cameroon; 2 Department of Internal Medicine, Douala General Hospital, PO Box: 4856, Douala, Cameroon; 3 Higher Institute of Health Sciences, Université des Montagnes, Bangangte, Cameroon; 4 Faculty of Medicine and Biomedical Sciences, University of Yaoundé I, Yaoundé, Cameroon; 5 Department of Internal Medicine, Yaounde General Hospital, Yaoundé, Cameroon

**Keywords:** Chronic kidney disease, Human immune deficiency virus, Douala, Cameroon

## Abstract

**Background:**

Chronic kidney disease (CKD) is one of the major complications of Human immune deficiency Virus (HIV) and a risk factor for poor outcome of these patients. We aimed to describe the profile and outcome of HIV positive patients with CKD in Douala general hospital in Cameroon.

**Methods:**

HIV positive patients with CKD referred to the nephrologist from January 2007 to March 2013 were included. Socio demographic, clinical (history and stage of HIV, comorbidities, baseline nephropathy, used of c-ART), para clinical data at referral (serum urea, creatinine, full blood count, CD4 count, serum calcium, phosphorus, albumin), dialysis initiation and outcome at 1 year were collected from medical records. GFR was estimated using Chronic Kidney Disease Epidemiology Collaboration (CKD-EPI) equations. CKD was defined and classified according to the *Kidney Disease Improving Global Outcomes (KDIGO 2012).*

**Results:**

We included 156 patients (51.3% men) with a mean age of 45.4 ± 12.1 years. Hypertension (36.5%), diabetes (17.9%) and Hepatitis C (7.7%) were the main comorbidities. HIV associated nephropathy (27.6%), chronic glomerulonephritis (15.4%) diabetes (14.1%) and hypertension (13.5%) were the leading causes of kidney disease. Before referral HIV status was known by 109 (69.9%) patients, with 76 (69.7%) being on c-ART. Median CD_4_ count was 241 (117–438) cells/mm^3^. Prevalence of anemia (93.9%), hypocalcemia (68.6%) and Proteinuria (77.6%) was high, 94 (60.3%) patients were at CKD stage 5 at referral and 37 (23.7%) underwent emergency dialysis. After 1 year, 64 (41.0%) patients were lost to follow up. The mortality rate was 49% and 25 (28.7%) were maintenance hemodialysis, and being on c-ART was associated with a lower risk of death (HR: 0.45; 95% CI: 0.23–0.89; *p* = 0.021).

**Conclusion:**

HIV patients with CKD were referred late with high morbidity and need for urgent hemodialysis. HIVAN was the main etiology of CKD and mortality rate was high mainly due to the absence of c-ART at referral.

## Background

The number of people living with human immunodeficiency virus (HIV) is increasing, with 36.9 million people infected in 2015 and 2 million news cases per year [1]. The introduction of combined antiretroviral therapy (c-ART) has improved the lifespan of HIV infected patients and exposed them to various organs damage [2]. Chronic kidney disease (CKD) is one of the major complications amongst HIV infected patients, with a prevalence ranging from 3.5 to 48.5% [2–5] and the presence of CKD is a risk factor for mortality of these patients [6–8].

CKD in HIV can be related to the virus itself or no. Common disease due to the virus are HIV associated nephropathy (HIVAN) and HIV-immune complex nephropathy (HIVICK). Black people from African origin are more prone to HIVAN due to predisposing genetic polymorphismus [9, 10] and the risk is 3 to 6 fold higher compared to white [11, 12]. HIVAN was the 3th leading cause of end stage kidney disease (ESKD) amongst black in the United States of America [13, 14]. The introduction of c-ART has reduced the incidence of HIVAN and ESKD [3, 15], but CKD remain a serious problem in these patients. Others HIV-related factors are low CD4 counts, high viral load, hepatitis C virus co-infection and some antiretroviral drugs such as Tenofovir, Indinavir, Lopinavir/Ritonavir, Atazanavir/Ritonavir [11, 16–19]. Other reported causes of CKD not HIV related are older age, diabetes mellitus and hypertension [4, 5, 7, 20, 21]. In a study of Jung et al. in Germany, HIVICK (26.1%), nephroangiosclerosis and diabetic nephropathy (20.3% respectively) were the leading causes of CKD in HIV positive patients [22] while Wyatt et al. and Onen et al. reported hypertensive renal disease as the most common kidney disease [23, 24]. In Spain, diabetic nephropathy was the etiology of ESKD in 14% of HIV-positive patients [25].

Early identification of kidney disease and early referral to the nephrologist gives a chance to implement treatments that slow the progression of kidney dysfunction and reduces the need of renal replacement therapy (RRT) [26, 27] and therefore reduces mortality of patients.

The prevalence and incidence of HIV in Sub-Saharan Africa (SSA) is the highest in the world (75.8% of new cases) with greatest mortality rate [28, 1]. Kidney disease is frequent amongst these patients in SSA with a pooled prevalence of 14.6% [29]. In Cameroon a country in SSA, HIV is the 5th leading cause of ESKD with a prevalence of 10.8 to 13.5% amongst patients on maintenance hemodialysis [30–32]. Late referral of CKD patients to the nephrologist is common in that setting and outcome of patients is poor in general and survival of HIV infected patients on RRT is lower compared to their negative counterpart [8, 33, 34]. Studies on HIV patients with non-dialyzed CKD are rare in SSA. The aim of this study was to describe the profile and outcome of HIV positive patients with CKD in Cameroon, a country where patients have free access to c-ART.

## Methods

### Study setting

We conducted a retrospective study in the nephrology outpatient consultation of the Douala general hospital, the main referral tertiary hospitals for patients with kidney disease in the littoral region with a capacity of 320 beds. At the time of the study, the center operated with one internist- nephrologist and two general practitioners. Each patient has a medical file that is opened at the first consultation. In Cameroon, c-ART is highly subsidized by the government since 2007and access to treatment is not limited. HIV patients with CKD are followed up by internist-nephrologist with a special training in management of HIV related diseases. The study was authorized by the Director of the Douala general hospital and ethical approval was obtained from the Douala University Ethics Committee.

### Patients

All HIV positive patients aged 18 years and above with CKD referred to the nephrologist from 1 January 2007 to 31th March 2013 were included. HIV patients with missing relevant data were excluded. Data were collected from medical records and included socio demographic (age, sex, marital status), clinical (history and stage of HIV, comorbidities, baseline nephropathy, used of c-ART before referral), para clinical data at referral (serum urea, creatinine, full blood count, CD4 count, serum calcium, phosphorus, albumin), dialysis initiation and outcome at 1 year (death, loss of follow up, alive, on dialysis).

### Definition of operational terms

Estimated glomerular filtration rate (e GFR, mL/min) was computed using Chronic Kidney Disease Epidemiology Collaboration (CKD-EPI) equations [35].

CKD was defined and classified according to the *Kidney Disease Improving Global Outcomes (KDIGO 2012)* [36]. eGFR was therefore classified as follow: G1 (eGFR ≥90); G2 (eGFR 60–89); G3a (eGFR 45–59); G3b (eGFR 30–44); G4 (eGFR 15–29) and G5 (eGFR < 15).

ESKD was considered in any patient with eGFR < 15 ml/min/1.73m^2^ and on maintenance hemodialysis for more than 3 months. Hypertension was defined as a blood pressure > 140/90mmhg or evidence from records that the patient was on antihypertensive treatment. Diabetes was considered if a reported history of diagnostic of this condition or use of glucose lowering l agents, or fasting blood sugar above 1.26 g/ml. The background nephropathy was made by the nephrologist and mainly based on clinical arguments in the absence of renal histology data.

HIVAN was considered if a CKD patient presented with nephrotic range proteinuria with or without edema, normal blood pressure, hyperechogenic kidney with normal size on ultrasound.

Lost to follow up was considered in any patient who was recorded at the start of the study period, and become lost within the first year of follow up, with no data on the outcome.

### Statistical analysis

Data were analyzed using the software Statistical Package for Social Sciences (SPSS Inc., Chicago, USA) version 23.0. Qualitative variables were described as frequency and percentages and compared with Chi-square test or Fisher’s exact test. Quantitative variables with normal distribution were described as mean ± standard deviation (SD); skewed data as median and inter quartile range (IQR). Their comparison was done respectively with Student T-test and Mann Whitney U test. One year mortality rate was calculated by dividing the number of deceased patients by the number of patients whose outcome was known at 1 year (alive or death). Predictors of death were assessed using Cox proportional hazard regression models. We adjusted the basic models for age and sex. Survival curves of patients treated or not with c-ART at referral were compared using Kaplan Meier estimator (Log rank test). Statistical significance was set at a *p* value < 0.05.

## Results

### Baseline characteristics

A total of 156 patients were included in this study with 80/156 (51.3%) men. Mean age of participants was 45.4 ± 12.1 years and women were younger (*p* = 0.002). Mean age wasn’t significantly different across stage of kidney disease (*p* = 0.39). Their baseline characteristics are shown in Table 1. Hypertension (36.5%), diabetes (17.9%) and hepatitis C (7.7%) were the main comorbidities. HIVAN 43/156 (27.6%), chronic glomerulonephritis 24/156 (15.4%) diabetes 22/156 (14.1%), and hypertension 21/156 (13.5%) were the leading causes of kidney disease. In 24.4% of cases the etiology was unknown. Before referral 109/156 (69.9%) patients knew their HIV status of which only 76/109 (69.7%) were already on c-ART. Median CD_4_ count was 241 (117–438) cells/mm^3^, with no difference between genders (*p* = 0.619) or CKD stage (*p* = 0.456).

**Table 1:** General characteristics of the study population at referral

Variables	Total	Gender		*P*	Stage				*P*
		Male	Female		G3a	G3b	G4	G5	
N (%)	156 (100.0)	80 (51.3)	76 (48.7)		13 (8.3)	20 (12.8)	32 (20.5)	91 (58.3)	
Age (years), Mean ± SD^a^	45.4 ± 12.1	48.3 ± 11.8	42.4 ± 11.6	*0.002*	46.6 ± 14.9	49.0 ± 12.8	43.2 ± 12.7	45.3 ± 11.2	0.394
Age (years), Min-Max	22–82	25–82	22–72		26–72	28–70	25–82	22–77	
Marital status, n (%)									
Married	87 (55.8)	58 (72.5)	29 (38.2)		9 (69.2)	9 (45.0)	17 (53.1)	52 (57.1)	
Single	53 (34.0)	17 (21.3)	36 (47.4)		3 (23.1)	8 (40.0)	13 (40.6)	29 (31.9)	
Divorced	2 (1.3)	1 (1.3)	1 (1.3)		0 (0.0)	0 (0.0)	1 (3.1)	1 (1.1)	
Widow	14 (9.0)	4 (5.0)	10 (13.2)	*< 0.001*	1 (7.7)	3 (15.0)	1 (3.1)	9 (9.9)	0.784
Employment status, n (%)									
Unemployed	56 (35.9)	11 (13.8)	45 (59.2)		6 (46.2)	4 (20.0)	12 (37.5)	34 (37.4)	
Public agent	10 (6.4)	8 (10.0)	2 (2.6)		0 (0.0)	1 (5.0)	1 (3.1)	8 (8.8)	
Private sector	37 (23.7)	29 (36.3)	8 (10.5)		5 (38.5)	5 (25.0)	8 (25.0)	19 (20.9)	
Informal sector	39 (25.0)	18 (22.5)	21 (27.6)		1 (77)	6 (300)	8 (25.0)	24 (26.4)	
Retired	14 (9.0)	14 (17.5)	0 (0.0)	*< 0.001*	1 (7.7)	6 (30.0)	8 (25.0)	24 (26.4)	0.548
Co morbidity, n (%)									
Hypertension	57 (36.5)	25 (31.6)	32 (42.1)	0.177	4 (33.3)	6 (30.0)	10 (31.3)	37 (40.7)	0.690
Diabetes	28 (17.9)	18 (22.8)	10 (13.2)	0.119	2 (16.7)	6 (30.0)	7 (21.9)	13 (14.3)	0.372
HCV^b^ coinfection	12 (7.7)	7 (8.8)	5 (6.6)	0.611	1 (7.7)	2 (10.0)	1 (3.1)	8 (8.8)	0.742
Chronic use of NSAID^c^	9 (5.8)	4 (5.1)	5 (6.6)	0.744	1 (8.3)	1 (5.0)	1 (3.1)	6 (6.6)	0.876
HBV^d^ coinfection	6 (3.8)	5 (6.3)	1 (1.3)	0.211	0 (0.0)	2 (10.0)	3 (9.4)	1 (1.1)	0.070
Background nephropathy, n (%)									
HIVAN^e^	43 (27.6)	26 (32.5)	17 (22.4)		4 (30.8)	5 (25.0)	6 (18.8)	28 (30.8)	
Chronic glomerulonephritis	24 (15.4)	9 (11.3)	15 (19.7)		1 (7.7)	5 (25.0)	5 (15.6)	13 (14.3)	
Diabetes	22 (14.1)	15 (18.8)	7 (9.2)		1 (7.7)	6 (30.0)	4 (12.5)	11 (12.1)	
Hypertension	21 (13.5)	7 (8.8)	14 (18.4)		3 (23.1)	2 (10.0)	12 (37.5)	21 (23.1)	
Chronic interstitial nephritis	7 (4.5)	3 (3.8)	4 (5.3)		1 (7.7)	0 (0.0)	1 (3.1)	5 (5.5)	
Polykystosis	1 (0.6)	1 (1.3)	0 (0.0)		0 (0.0)	1 (5.0)	0 (0.0)	0 (0.0)	
Unknown	38 (24.4)	19 (23.8)	19 (25.0)	0.139	3 (23.1)	2 (10.0)	12 (37.5)	21 (23.1)	0.242
Known HIV^e^ infected, n (%)	109 (69.9)	56 (70.0)	53 (69.7)	0.971	8 (61.5)	16 (80.0)	21 (65.6)	64 (70.3)	
HIV infection vintage (month)^g^	36 (11–96)	36 (12–108)	36 (10.3–96)	0.856	24 (1–144)	15 (4–48)	24 (7.5–60)	36 (12–108)	0.251
Use of cART^f^, n (%)	76 (48.7)	36 (45.0)	40 (52.6)	0.340	7 (53.8)	10 (50.0)	11 (34.4)	48 (52.7)	
Duration on cART^fg^	36 (6.8–96)	36 (12–99)	36 (6–96)	0.879	40 (18.3–90)	8 (2–79.5)	48 (4–72)	36 (9.8–105)	0.797
Drug regimen, n (%), *n* = 76									
1st line	65 (85.5)	33 (91.7)	32 (80.0)		6 (85.7)	8 (80.0)	8 (72.7)	43 (89.6)	
2^nd^ line	11 (14.5)	3 (8.3)	8 (20.0)	0.199	1 (14.3)	2 (20.0)	3 (27.3)	5 (10.4)	
CD_4_ count^g^, *n* = 88	241 (117–438)	256 (138–387)	216 (96–535)	0.619	101 (66–376)	176.5 (137–333.3)	278 (117.3–549)	271 (120–451)	0.456

Values in italics are significant (*p* value< 0.05)

^a^*SD* Standard deviation, ^b^*HCV* Hepatitis C virus, ^c^*NSAID* Non-steroidal anti-inflammatory drugs, ^d^*HBV* Hepatitis B virus, ^e^*HIVAN* Human immuno-deficiency virus associated nephropathy, ^f^*cART* Combined antiretroviral treatment; ^g^Median (1st-3th quartiles)

### Biological profile

Biologic data of study participants are reported in Table 2. Most patients (58.3%) had CKD stage 5 at referral. Blood Urea Nitrogen and Creatinine significantly increase with stage of CKD (*p* < 0.001). Median GFR was 10.1 ml/min/1.73m^2^ overall and was lower in women compare to men (*p* = 0.019) and decreases with stage of CDK (*p* < 0.001). Proteinuria was present in 121 (77.6%) patients, hematuria in 42 (26.9%), leucocyturia in 23 (14.7%) and glycosuria in 10 (6.4%) patients with no statistically difference between genders or stage of kidney disease. Mean hemoglobin level was 8.2 ± 2.3 g/dl overall and lower in women (*p* = 0.019) and decreases with stage of CKD (*p* = 0.004). Anemia was frequent with a prevalence of 93.9% (90.7% in men, 97.3% in women, *p* = 0.167) and was more common in late stage of CKD; from 75% in stage 3a to 97.7% in stage 5 (*p* = 0.017). Mean calcemia was 84.6 ± 13.7 mg/l and lower in women (*p* = 0.024) and did not varies significantly across stage of CKD (*p* = 0.413). Hypocalcemia concerned 70/102 (68.6%) with no difference neither across gender nor stage of disease. Phosphoremia increases with stage of CKD with a borderline *p* value (0.056). Hyperphosphoremia was seen in 32/89 (36.0%) patients and its frequency increases with stage of CKD (16.7% in stage 3a vs 45.2% in stage 5). The distribution of other hematologic parameters, electrolytes, lipid profile showed no major differences across gender or stage of CKD.

**Table 2:** Biologic profile and stage of chronic kidney disease of study participants at referral

Biologic Data	Total	Gender		*P*	Stage				*P*
		Male	Female		G3a	G3b	G4	G5	
Blood urea nitrogen (g/l)^h^	1.12 (0.7–2.1)	1.1 (0.7–2.0)	1.3 (0.7–2.2)	0.918	0.4 (0.3–0.6)	0.7 (0.5–1.2)	0.8 (0.6–1.1)	1.8 (1.05–2.5)	*< 0.001*
Serum Creatinine (mg/l)^h^	67.7 (29.3–118.5)	73.4 (29.6–101.3)	61.9 (28.4–135.1)	0.457	16.7 (15.8–19.6)	24.7 (21–25.4)	33.9 (29.6–40.6)	103 (79.8–160.9)	*< 0.001*
GFR^a^ (ml/min/1.73 m^2^)^h^	10.1 (4.7–27.2)	10.2 (6.5–31.5)	9.5 (3.4–23.3)	*0.028*	49.5 (47.9–51.8)	37.1 (31.9–40.5)	22.4 (19.8–26.51)	5.3 (3.2–8.3)	*< 0.001*
CKD^b^ stage, n (%)					–	–	–	–	
G1	0 (0.0)	0 (0.0)	0 (0.0)		–	–	–	–	
G2	0 (0.0)	0 (0.0)	0 (0.0)		–	–	–	–	
G3a	13 (8.3)	8 (10.0)	5 (6.6)		–	–	–	–	
G3b	20 (12.8)	13 (16.3)	7 (9.2)		–	–	–	–	
G4	32 (20.5)	17 (21.3)	15 (19.7)		–	–	–	–	
G5	91 (58.3)	42 (52.5)	49 (64.5)	0.383	–	–	–	–	
Urinary dipstick, n (%)									
Proteinuria	121 (77.6)	65 (81.3)	56 (73.7)	0.258	7 (53.8)	15 (75.0)	27 (84.4)	72 (79.1)	0.154
Hematuria	42 (26.9)	26 (32.5)	16 (21.1)	0.107	2 (15.4)	5 (25.0)	5 (15.6)	30 (33.3)	0.183
Leucocyturia	23 (14.7)	10 (12.5)	13 (17.1)	0.417	1 (7.7)	3 (15.0)	3 (9.4)	16 (17.8)	0.594
Glycosuria	10 (6.4)	5 (6.3)	5 (6.6)	0.933	0 (0.0)	1 (5.0)	2 (6.3)	7 (7.8)	0.746
Lipid profile, Mean ± SD									
Total cholesterol (g/l)	1.8 ± 0.6	1.7 ± 0.5	2.0 ± 0.7	0.109	1.7 ± 0.3	1.9 ± 0.5	2.1 ± 0.7	1.8 ± 0.6	0.551
LDL^c^ cholesterol (g/l)	1.0 ± 0.6	1.0 ± 0.6	1.1 ± 0.7	0.668	0.4 ± 0.2	0.3 ± 0.2	0.5 ± 0.2	0.4 ± 0.2	0.214
HDL^d^ cholesterol (g/l)	0.4 ± 0.2	0.3 ± 0.1	0.4 ± 0.2	0.064	0.6 ± 0.2	1.0 ± 0.1	1.1 ± 0.7	1.0 ± 0.7	0.825
Triglycerides (g/l)	1.8 ± 1.1	1.6 ± 0.8	2.0 ± 1.3	0.317	2.3 ± 0.2	1.6 ± 2.1	1.7 ± 0.9	1.9 ± 0.9	0.857
Hemoglobin (g/dl), Mean ± SD	8.2 ± 2.3	8.7 ± 2.4	7.6 ± 1.9	*0.002*	9.8 ± 3.3	9.0 ± 2.2	8.5 ± 2.2	7.7 ± 2.0	*0.004*
Anemia, *n* = 148, n (%)	139 (93.9)	68 (90.7)	71 (97.3)	0.167	9 (75.0)	18 (90.0)	29 (93.5)	84 (97.7)	*0.017*
MCV^e^ (fl), *n* = 107									
Mean ± SD	85.5 ± 9.8	85.9 ± 9.4	85.1 ± 10.2	0.674	85.5 ± 9.8	85.8 ± 8.1	85.3 ± 7.9	85.4 ± 10.7	0.999
< 80	34 (31.8)	17 (31.5)	17 (32.1)		3 (50.0)	4 (30.8)	6 (30.0)	21 (30.9)	
> 100	8 (7.5)	4 (7.4)	4 (7.5)	0.997	0 (0.0)	0 (0.0)	1 (5.0)	7 (10.3)	0.765
MCH^f^ (pg), *n* = 100									
Mean ± SD	28.1 ± 4.3	28.0 ± 5.0	28.1 ± 3.5	0.885	29.2 ± 3.6	28.6 ± 3.8	27.6 ± 3.3	28.3 ± 4.0	0.791
< 28	48 (48.0)	22 (45.8)	26 (50.0)		3 (50.0)	6 (46.2)	11 (57.9)	28 (45.2)	
> 32	15 (15.0)	8 (16.7)	7 (13.5)	0.875	2 (33.3)	2 (15.4)	2 (10.5)	9 (14.5)	0.793
WBC^g^ (G/l)^h^, *n* = 124									
Median (1st-3th quartiles)	5.3 (4.1–7.1)	5.3 (4.4–7.2)	5.1 (3.9–7.1)	0.276	4.9 (4.2–5.3)	5.3 (3.8–8.8)	5.2 (4–8)	5.4 (4.1–7.1)	0.843
< 4	25 (20.7)	9 (15.0)	16 (26.2)		0 (0.0)	5 (31.3)	5 (21.7)	15 (20.3)	
> 10	12 (9.9)	5 (8.3)	7 (11.5)	0.218	0 (0.0)	1 (6.3)	1 (4.3)	10 (13.5)	0.314
Platelets (G/l), *n* = 121									
Mean ± SD	223.4 ± 94.1	214.6 ± 93.0	232.4 ± 95.2	0.300	224.3 ± 112.5	213.5 ± 85.4	214.4 ± 91.6	223.6 ± 93.6	0.877
< 150	25 (20.7)	16 (26.2)	9 (15.0)	0.267	2 (22.2)	2 (11.1)	5 (20.8)	16 (22.5)	0.511
Natremia (mmol/l), *n* = 111									
Mean ± SD	135.0 ± 8.8	134.6 ± 9.1	135.4 ± 8.6	0.639	136.1 ± 6.5	134.9 ± 6.1	136.8 ± 10.1	134.3 ± 9.2	0.718
< 135	44 (39.6)	23 (40.4)	21 (38.9)	0.571	5 (55.6)	5 (38.5)	4 (19.0)	31 (44.9)	0.393
Potassium (mmol/l), *n* = 109									
Mean ± SD	5.3 ± 3.3	5.0 ± 0.98	4.9 ± 1.2	0.726	4.2 ± 0.5	4.8 ± 0.9	4.8 ± 0.8	5.1 ± 1.2	0.096
< 3.5	4 (3.7)	2 (3.6)	2 (3.7)		0 (0.0)	0 (0.0)	1 (5.0)	3 (4.3)	
> 5.0	47 (43.1)	27 (49.1)	20 (37.0)	0.437	1 (11.1)	6 (50.0)	10 (50.0)	30 (43.5)	0.435
Chloremia (mmol/l), *n* = 101									
Mean ± SD	101.7 ± 9.4	101.0 ± 9.7	102.6 ± 8.9	0.410	99.1 ± 10.3	103.8 ± 11.5	103.1 ± 7.2	101.3 ± 9.4	0.631
< 95	25 (24.8)	14 (25.5)	11 (23.9)		3 (37.5)	3 (25.0)	3 (17.6)	16 (24.6)	
> 105	33 (32.7)	15 (27.3)	18 (39.1)	0.421	2 (25.0)	6 (50.0)	5 (29.4)	20 (30.6)	0.724
Calcemia (mg/l), *n* = 102									
Mean ± SD	84.6 ± 13.7	87.5 ± 15.3	81.5 ± 11.1	*0.024*	84.6 ± 8.8	89.9 ± 10.5	87.5 ± 10.0	83.1 ± 15.1	0.413
< 90	70 (68.6)	31 (60.8)	39 (76.5)		5 (62.5)	5 (55.6)	8 (47.1)	52 (75.4)	
> 105	2 (2.0)	2 (3.9)	0 (0.0)	0.128	0 (0.0)	1 (11.1)	0 (0.0)	1 (1.4)	0.102
Phosphoremia (mg/l), *n* = 89									
Mean ± SD	56.6 ± 31.5	50.8 ± 26.4	62.3 ± 35.1	0.086	38.2 ± 19.3	36.6 ± 8.5	49.9 ± 28.2	62.1 ± 33.2	0.056
< 35	22 (24.7)	12 (27.3)	10 (22.2)		2 (33.3)	5 (71.4)	5 (35.7)	10 (16.1)	
> 55	32 (36.0)	11 (25.0)	21 (46.7)	0.096	1 (16.7)	0 (0.0)	3 (21.4)	28 (45.2)	*0.022*
Uricemia (mg/l), *n* = 66									
Mean ± SD	89.3 ± 32.2	91.7 ± 33.2	88.7 ± 31.4	0.530	78.1 ± 16.1	78.5 ± 24.2	88.4 ± 35.2	93.8 ± 34.2	0.509
> 70	50 (75.8)	27 (79.4)	23 (71.9)	0.515	5 (83.3)	6 (75.0)	11 (73.3)	28 (75.7)	0.982
Albuminemia (g/l), *n* = 80									
Mean ± SD	32.8 ± 11.2	33.5 ± 13.9	31.9 ± 7.5	0.531	34.0 ± 2.9	29.7 ± 7.2	37.3 ± 18.4	31.7 ± 9.0	0.294
< 35	51 (63.7)	27 (65.9)	24 (61.5)	0.688	3 (60.0)	6 (75.0)	9 (56.3)	34 (65.4)	0.822

Values in italics are significant (*p* value< 0.05)

^a^*GFR* Glomerular filtration rate (estimated using MDRD formulae), ^b^*CKD* Chronic kidney disease, ^c^*LDL* Low density lipoprotein, ^d^*HDL* High density lipoprotein, *MCV*^e^ Mean corpuscular volume, *MCH*^f^ Mean corpuscular haemoglobin, *WBC*^g^ White blood cells; ^h^Median (1st-3th quartiles)

### Outcome

In total, 37/156 (23.7%) patients initiated dialysis immediately at referral and 34/37 (91.9%) on a temporary central venous catheter. Indication for dialysis were: uremic syndrome (81.1%), acute pulmonary edema (16.2%), hyperkalemia (10.8%), and uremic encephalopathy (8.1%) (Table 3). After 1 year of follow up, 64 patients (41%) were lost to follow up and 5 (3.2%) were transferred to another nephrology unit (Table 3). For those with known outcome at 1 year (87/156) the overall mortality rate was 49.4% (43/87 patients) and was similar between both sex (*p* = 0.914), and 19 (21.8%) patients were on chronic hemodialysis. As shown in Table 4, outcome after 1 year of follow up varied according to the stage of kidney disease with respectively 40, 33.3, 45.5 and 15.4% of patient of stage 3a, 3b, 4 and stage 5 being alive and not on chronic dialysis with a borderline *p* value (0.085). The rate of patients lost to follow up varied across CKD stages: stage 3a (61.5%), 3b (70.0%) and 4 (62.5%) than in stage 5 (24.2%) of disease (*< 0.001)*.

**Table 3:** Outcome of the study population after 1 year of follow up

Outcome	Total n (%) *N* = 156	Male n (%) *N* = 80	Female n (%) *N* = 76	*P*
At referral				
Dialysis	37 (23.7)	16 (20.0)	21 (27.6)	0.263
Vascular access				
*CVC*^*a*^	34 (91.9)	16 (100.0)	18 (85.7)	
*AVF*^*b*^	3 (8.1)	0 (0.0)	3 (14.3)	0.243
Dialysis indication, *n* = 37				
Uremic syndrome	30 (81.1)	12 (75.0)	18 (85.7)	0.437
Acute pulmonary edema	6 (16.2)	3 (18.8)	3 (14.3)	> 0.999
Severe hyperkalemia	4 (10.8)	212.5)	2 (9.5)	> 0.999
Uremic encephalopathy	3 (8.1)	1 (6.3)	2 (9.5)	> 0.999
At 1 year				
Unknown				
Transfer to another hospital	5 (3.2)	3 (3.8)	2 (2.6)	0.692
Lost to follow up	64 (41.0)	34 (42.5)	30 (39.5)	0.701
Known, *n* = 87				
Deceased	43 (49.4)	21 (48.8)	22 (50.0)	0.914
On chronic dialysis	25 (28.7)	12 (27.9)	13 (29.5)	0.866
Alive and not on dialysis	19 (21.8)	10 (23.3)	9 (20.5)	0.752
CD4^c^, *n* = 21	380 (265–598.5)	353.5 (251.5–588.8)	476 (271.5–685)	0.804

^a^*CVC* Central venous catheter, ^b^*AVF* Arteriovenous fistulae; ^c^Median (1st-3th quartiles)

**Table 4:** Outcome by stage of kidney disease after 1 year of follow up

Outcome	Stage of kidney disease				*P*
	G3a *N* = 13	G3b *N* = 20	G4 *N* = 32	G5 *N* = 91	
Unknown					
Transfer to another hospital	0 (0)	0 (0)	1 (3.1)	4 (4.4)	0.680
Lost to follow up	8 (61.5)	14 (70.0)	20 (62.5)	22 (24.2)	*< 0.001*
Known	*n* = 5	*n* = 6	*n* = 11	*n* = 65	
Deceased	2 (40.0)	3 (50.0)	4 (36.4)	34 (52.3)	0.766
On chronic dialysis	1 (20.0)	1 (16.7)	2 (18.2)	21 (32.3)	0.656
Alive and not on dialysis	2 (40.0)	2 (33.3)	5 (45.5)	10 (15.4)	0.085

Values in italics are significant (*p* value< 0.05)

**Fig. 1:**
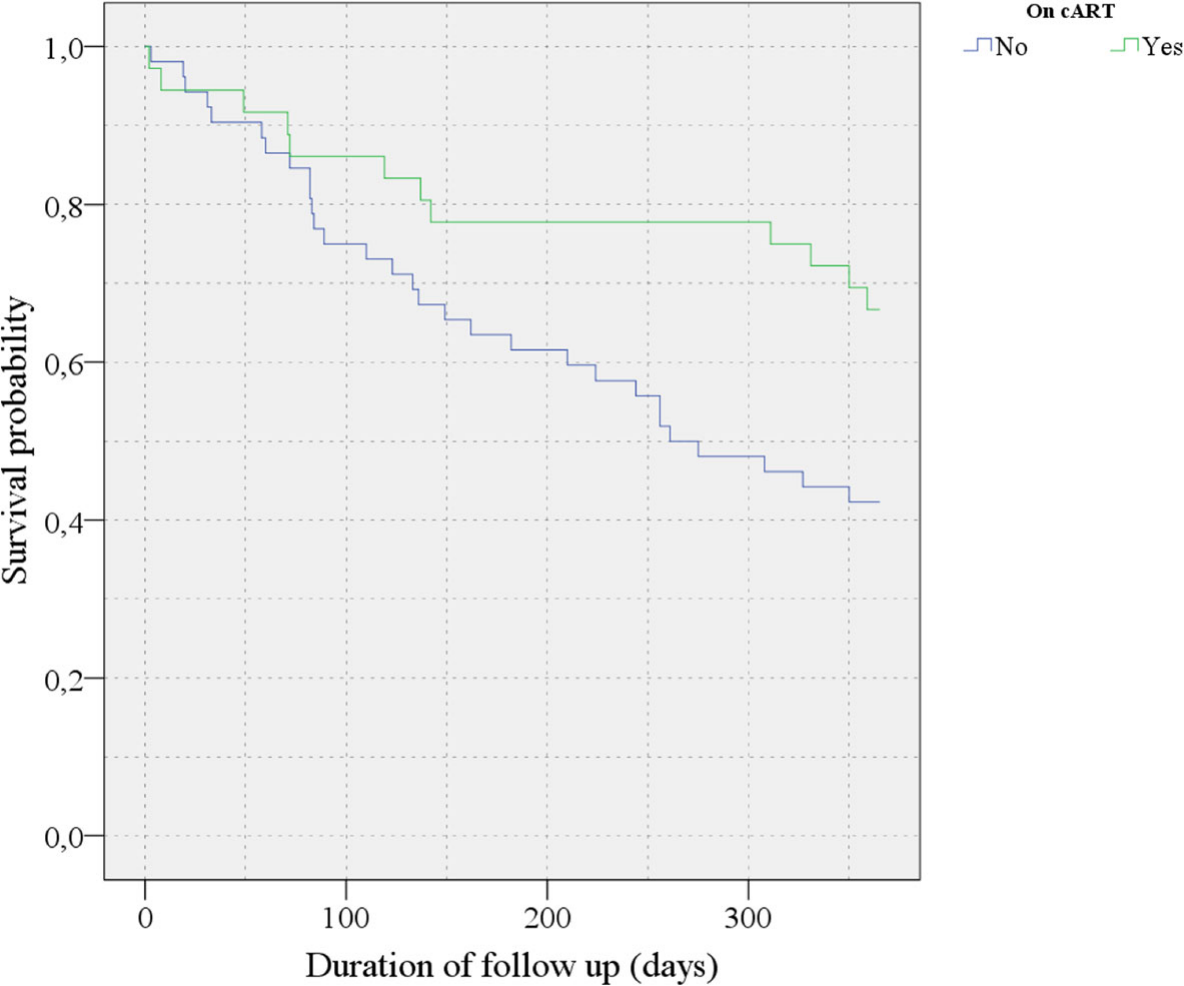
Survival probability during follow-up from Kaplan Meier estimator comparing patients treated with (blue curve) and without (green curve) c-ART at referral; *p* value = 0.021 for Log Rank test

Age and sex adjusted Cox regression analysis showed that being on c-ART at referral was associated with a lower risk of death in the first year of follow up; Hazard Ratio: 0.45; 95% CI: 0.23–0.89; *p* = 0.021 (Table 5). Kaplan Meier estimator confirm this observation, with a better survival curve in patients already on c-ART at referral. (*p* = 0.021 for Log Rank test) Fig. 1.

**Table 5:** Predictive factors of mortality (Cox regression analysis)

Variable	Basic models		Final models	
	HR^a^ (95% CI^b^)	*p*	HR^a^ (95% CI^b^)	*p*
Age (per years increase)	0.99 (0.96–1.02)	0.452	1.00 (0.97–1.02)	0.742
Gender (female vs male)	1.02 (0.55–1.89)	0.963	1.15 (0.61–2.16)	0.663
Unemployed	1.37 (0.68–2.73)	0.380		
Hypertension	1.58 (0.84–2.96)	0.156		
Diabetes	0.56 (0.23–1.40)	0.216		
Hepatitis B	0.61 (0.08–4.53)	0.632		
Hepatitis C	1.43 (0.58–3.55)	0.442		
On cART^c^	0.45 (0.23–0.89)	*0.021*	0.45 (0.23–0.89)	*0.021*
CD4 count (cells/mm^3^)	1.00 (0.99–1.00)	0.823		
Stage of CKD at arrival				
G3	1 (reference)			
G4	0.45 (0.10–2.03)	0.299		
G5	1.16 (0.42–3.17)	0.780		
Hemoglobin level (per g/dl)	0.91 (0.79–1.05)	0.201		
Calcium level (per 10 mg/l)	0.81 (0.59–1.10)	0.178		
Creatinine level (per 10 mg/l)	1.01 (0.97–1.05)	0.606		

Basic models are adjusted for age and sex; final models are adjusted for age, sex and all predictors with a *p* value < 0.1 in the basic models (use of cART)

Values in italics are significant (*p* value< 0.05)

^a^*HR* Hazard ratio, ^b^*CI* Confidence interval, ^c^*cART* Combined anti-retroviral treatment

## Discussion

This study on the characteristic of HIV positive patients with CKD referred to a tertiary hospital in SSA showed that included patients were young adults, with others risks factors of CKD such as hypertension, diabetes and HVC. Proteinuria was the main urinary abnormality affecting more than ¾ of patients. At referral median CD4 was low and less than half of the study population were on c-ART. HIVAN was the main presumed etiology of CKD. Patients were referred at advanced stage of CKD for the majority with various biological abnormalities among witch anemia was the most frequent. Dialysis was initiated in almost ¼ patients at referral due to life threatening conditions. At 1 year, the rate of loss of follow up and mortality was very high and absence of c-ART at referral was the main predictor of death.

CKD is epidemic among HIV-infected populations especially in Africa [29]. The prevalence of HIV in SSA is the highest in the world and kidney disease is frequent amongst these patients with a pooled prevalence of 14.6% [28, 29]. In the present study patients were young adult with mean age of 45 years and males represented half of the study population. It’s well known that CKD and HIV affects more young adults in SSA [37–39]. Contrary to our finding CKD affect more male due to genetic and environmental factor. One explanation to our finding is that HIV is more prevalent in women in our setting and therefore increase the number of women with CKD [37, 39, 40].

HIV infected patients often have one or more risk factors for CKD [7, 41, 42]. In the present study others non-HIV related risk factors were hypertension, diabetes, Hepatitis B and C infection. HIVAN was the leading cause of CKD (27.6%). This is in accordance with most studies in populations from African origin [38, 39, 43, 44]. Our reported rate is similar to the results of Okpechi et al. in South Africa and Da Silva et al. in Brazil [45, 46]. In the contrary Jung et al. in Germany found that HIVICK (26.1%) was the main etiology of CKD in HIV patients. This is due to racial difference as it is well known that HIVICK affect more white people [47]. The introduction of c-ART has reduced the incidence of HIVAN and ESKD in the world [15]. In Cameroon, patients have free access to c-ART. The prevalence of HIVAN found in this study remain high and possible raisons could be: the ignorance of the HIV status (30.1% at referral), the severity of the immune depression at referral (low median CD4 count), and the mostly the absence of c-ART for the majority of patients (51.3%) at referral. Others presumed etiology of CKD in this study were diabetes, chronic glomerulonephritis and hypertension. This is in accordance with reported studies [7, 20–25].

Our patients were referred at advanced stage of CKD with 60% at stage 5 with various clinical and biological complications. Consequently urgent dialysis without preparation was initiated in some patients at referral. Late referral is a serious problem in developing countries in general and in Cameroon in particular with high morbidity, emergency dialysis and poor outcome [33, 34, 48]. Early identification of kidney disease and early referral to the nephrologist gives a chance to implement treatments that slow the progression of kidney dysfunction and reduce the need of RRT and mortality of patients [26, 27].

We found at 1 year that 41% of patients were lost to follow up. This is extremely high and a dangerous situation. It is recommended that patients should have a regular nephrology care when e GFR < 30 ml/min/ 1.73 [36]. Raisons for this situation in our setting could be: the silent evolution of CKD as many patients visit the hospital only when they feel symptoms. Also the lack of funds is a serious problem; 35% of our patients were unemployed in a country where medical insurance is almost inexistent and the cost of care high and out-of-pocket spending represent an important proportion [49]. Also the fear of dialysis is common.

HIV and CKD are two risk factor that expose affected patients to death [50, 51] For those with known outcome at 1 year in this study (87/156) the overall mortality rate was high (49%). This rate is higher compare to mortality of ESKD patients in the same setting [8, 34] Studies have reported renal dysfunction as predictor of death amongst HIV infected patients and the risk increases with the stage of CKD [51, 52]. The main factor associated to death of our patients was the absence of c-ART at referral. This is in accordance with reported finding in the literature [15, 53, 54]. Similarly we reported recently in the same setting a lower survival rate of HIV patients on hemodialysis compared to negative one and the absence of c-ART was the main factor associated to death [8]. The use of c-ART and suppression of HIV RNA improved kidney function [55] and therefore reduce the risk of death. Studies have reported that earlier stage of the infection, higher CD4 counts, and treatment with c-ART was associated with better survival of HIV positive patients. [50, 56] Our patients were referred late. Current guidelines recommend specialist referral for patients with eGFR < 30 ml/min/ 1.73 m^2^ [36]. Absent or late pre dialysis nephrology care is associated with increased morbidity and mortality [33, 57, 58]. There is a need in our setting to evaluate the determinants of referral pattern and develop strategies to improve the situation.

### Limitations

We acknowledge some limitations to this study: first the retrospective design of the study with analysis based on data found in records. The absence of renal biopsy for the histological confirmation of the background nephropathy especially of HIVAN. But histological confirmation of HIVAN is infrequent in most studies from SSA and the diagnosis is usually made clinically. We did not have viral load and CD4 count for most patients during follow up, due to financial constraint as those test could are expensive and payment is out of pocket. Therefore the evaluation of control of the HIV infection was not possible. Also causes of death were not available. Data were collected from a single center, which could raise issues regarding the generalizability to the entire country. However, the study center is the only public institution to provide sufficient data for the range of time covered in the current study. This study has as major strength that it describe for the first time the baseline characteristics and outcome of HIV infected patients with CKD in Cameroon and in SSA. This could help to develop strategies to improve the care of these group of patients.

## Conclusion

This study revealed that HIV infected patients with CKD are mostly young adult, they are referred at late stage of the disease, with high morbidity and need for urgent hemodialysis. HIVAN is the main etiology of CKD and mortality rate is high. Absence of c-ART at referral might be associated with an increase in patient’s mortality. There is need to develop strategies for more multidisciplinary care of HIV infected patients and consequently improve their outcome.

## Data Availability

The datasets generated and/or analyzed during the current study are available from the corresponding author on reasonable request.

## Abbreviations

c-ART: Combined antiretroviral therapy
CKD: Chronic kidney disease
eGFR: Estimated glomerular filtration rate
ESKD: End stage kidney disease
HIV: Human immunodeficiency virus
HIVAN: HIV associated nephropathy
HIVICK: HIV-immune complex nephropathy
IQR: Inter quartile range
RRT: Renal replacement therapy
SD: Standard deviation
SSA: Sub-Saharan Africa
